# Postsurgical detection of glioma recurrence using MRI radiomics

**DOI:** 10.1093/noajnl/vdag143

**Published:** 2026-06-10

**Authors:** Daniel Abler, Bertrand Pouymayou, Jason M Keller, Adrien Depeursinge, Andrea Bink

**Affiliations:** Department of Oncology, Geneva University Hospitals and University of Geneva, Geneva, Switzerland; Department of Neuroradiology, Clinical Neuroscience Center, University Hospital Zurich, University of Zurich, Zurich, Switzerland; Department of Neuroradiology, Clinical Neuroscience Center, University Hospital Zurich, University of Zurich, Zurich, Switzerland; Institute of Informatics, University of Applied Sciences Western Switzerland (HES-SO), Sierre, Switzerland; Department of Nuclear Medicine and Molecular Imaging, Lausanne University Hospital, Lausanne, Switzerland; Lundin Family Brain Tumour Research Centre, Lausanne University Hospital, Lausanne, Switzerland; Department of Neuroradiology, Clinical Neuroscience Center, University Hospital Zurich, University of Zurich, Zurich, Switzerland

**Keywords:** glioma, machine learning, MRI, postoperative, post-therapeutic, radiomics

## Abstract

**Background:**

In the postoperative setting, distinguishing between treatment-related changes and glioma recurrence remains a major challenge in neuroradiology. This pilot study explored a dedicated radiomics-based approach applied to postoperative MRI.

**Methods:**

We retrospectively analyzed postoperative MRI scans from 38 patients with diffuse adult-type gliomas treated at the University Hospital Zurich. Recurrence was confirmed histologically or by clinico-radiological consensus. New contrast-enhancing VOIs were manually delineated on T1c images, and corresponding radiomics features were extracted from T1c and FLAIR images and were aggregated across VOIs. Logistic regression models were trained and evaluated on repeated random splits, with feature importance assessed through univariable analyses and selection frequency across iterations.

**Results:**

Feature aggregation by minimum value (min) and selection by maximum volume (maxVol) produced significantly predictive models for both FLAIR- and T1c-derived features. Shape features achieved significantly predictive performance, with AUCs of 0.78 [0.70, 0.85] (FLAIR) and 0.76 [0.70, 0.81] (T1c) on internal testing. Among individual features, Shape Sphericity showed the highest discriminative ability, distinguishing recurrence with AUCs of 0.79 (maxVol aggregation) and 0.82 (min aggregation).

**Conclusion:**

We identified robust shape-based radiomics features on T1c imaging that were significantly predictive of glioma recurrence. Dominant features—including Sphericity, Volume, and Surface-to-Volume Ratio—were consistently selected in 70% to 100% of modeling repetitions. Pending prospective validation in larger multi-institutional cohorts, these findings may ultimately support more accurate clinical decision-making in postoperative glioma management.

Key Points

- Postoperative MRI radiomics flags glioma recurrence.
- Radiomics shape features are most predictive of glioma recurrence.
- The dominant features were Sphericity, Volume, and Surface Volume Ratio.

Importance of the Study

This study addresses a common yet underexplored scenario: guiding clinical management decisions for individual patients when glioma recurrence is suspected on routine postoperative MRI. While most radiomics research has focused on predicting recurrence from preoperative images, little attention has been given to this postoperative context, where management decisions are often complex and resource-intensive.

We identified a group of common radiomics features that characterize the shape of imaging anomalies on contrast-enhanced T1-weighted (T1c) MR imaging and that were significantly predictive of tumor recurrence. The dominant features, Sphericity, Volume, and Surface Volume Ratio, were consistently selected in 70% to 100% of model iterations, highlighting their robustness and reproducibility.

Importantly, our approach provides complementary information to conventional radiological evaluation. When both radiological evaluation and radiomics signatures indicate suspicion of recurrence, our approach could provide stronger justification for costly and resource-intensive examinations such as FET-PET-MRI. Conversely, if the radiomics model suggests a low likelihood of recurrence, this may support a strategy of close routine monitoring with earlier follow-up MRI, potentially conserving financial and personnel resources.

Gliomas account for about 22% of all primary brain tumors and other central nervous system (CNS) tumors.^1^ The therapeutic management of gliomas, particularly glioblastomas, remains a significant challenge. Multidisciplinary approaches combining neurosurgery, neuro-oncology, radiation oncology, and neuroradiology aim at improving patient survival outcomes. Neuroradiological assessment with magnetic resonance imaging (MRI) before surgery, in certain patients during surgery (intraoperative MRI), and postoperatively is critical in the treatment pathway of glioma patients. Regular MRI follow-ups are performed for early detection of recurrence to react with treatment modification as early and as suitably as possible.

A major challenge during regular follow-up is the detection of imaging changes at a very early stage of recurrence, particularly when typical signs of recurrence are not yet measurable by MRI-perfusion. In such follow-up situations, the tumor board may decide to perform O-(2-[18F]fluoroethyl)-L-tyrosine positron emission tomography (FET PET) imaging. This imaging modality can help confirm or exclude tumor progression by analyzing tracer uptake patterns. However, its high cost and the associated additional radiation exposure limit its routine use and reduce accessibility.

In the field of artificial intelligence and imaging research, radiomics approaches have been employed to identify glioma recurrence using a variety of inclusion criteria, imaging methods (eg, MRI, PET), MRI sequences, and objectives. While the majority of studies have focused on predicting recurrence from pre-operative images,^2^^,^^3^ few have addressed the identification of recurrence using postoperative imaging. This task is particularly challenging due to the presence of the resection cavities and scarcity of tumoral tissue that is conventionally used as a region of interest (ROI) for radiomics feature extraction. Shahzadi et al^4^ applied deep radiomics in methionine (MET)-PET and MRI to predict both the prognosis and the presence of residual tumor after resection. ROIs were based on non-physiological MET-PET uptakes, and deep MET-PET radiomics achieved an area under the curve (AUC) of 0.95 for residual tumor detection. Ren et al^5^ applied conventional radiomics to posto­perative MRI to distinguish true tumor recurrence from treatment-related effects, with the best-performing model achieving an AUC of 0.97. Regions of interest included areas of edema and postoperative contrast enhancement, encompassing image anomalies resulting from prior treatments. However, the extraction of radiomic features and the prediction of true tumor recurrence from newly appearing imaging anomalies in the presence of resection cavities were not developed, although such an approach would better reflect typical clinical follow-up scenarios. While the results are very promising from a clinical perspective, to date, the identified radiomics features or signatures have not yet been translated into routine clinical decision-making.

In this study, we retrospectively included glioma patients with a neuroradiological suspicion of recurrence, for whom a FET-PET-Imaging report was available indicating the presence or absence of recurrence. Our primary objective was to evaluate whether MRI-based radiomics alone could provide valuable information in the assessment of suspected recurrence. Such information could support tumor board decisions, either by justifying a short-term MRI follow-up or by prompting FET-PET-Imaging to guide further treatment decisions.

## Methods

### Study Design and Patient Selection

This study was designed as a retrospective analysis, and no additional examinations or interventions were performed on the patients as part of this research. It was conducted in accordance with the principles of the Declaration of Helsinki. The retrospective evaluation of data from University Hospital Zurich was approved by the local ethics committee of the University of Zurich (Cantonal Ethic Committee, BASEC 2019-01862).

This study included 38 glioma patients (24 male, 14 female) aged between 20 and 75 years whose MRI scans were performed between 2015 and 2021. We selected patients who had undergone primary surgery, presented with a radiological suspicion of tumor recurrence on MRI, and subsequently received FET-PET-Imaging (34 FET-PET/MRI, 4 FET-PET/CT). Recurrence in this study was defined retrospectively based on established criteria: a ≥25% increase in the sum of the products of perpendicular diameters of enhancing lesions, or the appearance of any new lesions.^6^ Among the 21 patients with both MRI- and FET-PET-based suspicion of recurrence, 17 had histologically confirmed recurrence. The remaining 4 patients were not reoperated but were treated with chemotherapy, with recurrence diagnosed based on clinical and radiological progression. In contrast, 17 patients showed radiological suspicion of recurrence on MRI, but FET-PET imaging findings did not confirm recurrence. These patients were not reoperated on and showed no clinical or radiological evidence of recurrence during follow-up.

For detailed patient characteristics, please refer to Tables A1 and A2 in the Appendix. This study adheres to the reporting recommendations of the CheckList for EvaluAtion of Radiomics research (CLEAR),^7^ see Appendix A5.

### Image Acquisition

All examinations were performed on 3T MRI (Siemens or Philips) using varying scanning protocols. We analyzed the T1-weighted, T2-weighted, T1-weighted postcontrast (T1c), and FLAIR sequences.

The T1c in-plane pixel size ranged from 0.47 to 0.68 mm, with minimal variation between the groups (recurrence group: 0.54 ± 0.08 mm; non-recurrence group: 0.57 ± 0.1 mm). Slice thickness varied from 0.50 to 1.05 mm and was also comparable between the groups (recurrence: 0.87 ± 0.14 mm; non-recurrence: 0.83 ± 0.14 mm). Most images were acquired in the transversal plane, with 86% in the recurrence group and 88% in the non-recurrence group.

For the FLAIR images, in-plane pixel size ranged from 0.45 to 1.08 mm, again showing minimal variation between groups (recurrence group: 0.59 ± 0.19 mm; non-recurrence group: 0.64 ± 0.23 mm). Slice thickness ranged from 0.60 mm to 1.10 mm and remained comparable between the groups (recurrence: 0.89 ± 0.12 mm; non-recurrence: 0.85 ± 0.14 mm). Regarding imaging planes, 62% of images in the recurrence group and 65% in the non-recurrence group were acquired in the transversal plane.

### Image Processing and Extraction of Radiomics Features

For this study, the T1c MRI images served as a reference image for segmenting suspected tumor recurrences. In line with the aforementioned recurrence criteria [6], only tissue presumed to represent recurrent tumor was segmented, defined as newly enhancing tissue contiguous with prior residual tumor or enhancing tissue in a new location. This resulted in the creation of one or more 3D regions of interest (ROIs) per patient.

Segmentation of these ROIs was performed by a medical master student (MMS) under the supervision of a senior neuroradiologist (SN) with over 20 years of experience in neuro-oncological imaging analysis. Initially, the MMS and SN reviewed the images together to define the segmentation boundaries on the T1c sequences. The MMS then conducted the contouring, which was subsequently reviewed by the SN and, if necessary, corrected by the MMS until final approval by the SN was obtained. The segmentation process was performed using 3D Slicer (version 5.4.0).^8^

Following ROI segmentation, all MRI sequences were co-registered to the T1c reference sequence. DICOM (DCM) images and corresponding segmentations were then converted to NIfTI (NII) format for further processing. To standardize image intensity, six spherical ROIs (10 mm in diameter) were placed in normal-appearing white matter (WM) regions on the T1-weighted images. Z-score normalization was then applied, using the mean and standard deviation of intensities within these white matter ROIs to normalize each image. Images and segmentations were subsequently resampled to a standard resolution of 1 × 1 × 1 mm³ using linear and nearest neighbor interpolation for images and segmentation masks, respectively.

A total of 107 radiomics features (14 shape, 18 intensity, 75 texture) were extracted for each ROI and from each intensity-standardized MR sequence (T1, T1c, T2, FLAIR) using the Pyradiomics (version 2.4.4) library^9^ with standard settings (3D, “original” without filters). To ensure consistent Intensity quantization across patients and sequences, fixed bin widths were applied such that approximately 32 bins covered the range of standardized intensities for each sequence. The bin widths were set to 0.5 for T1, 0.8 for T1c, 1.0 for T2, and 0.8 for FLAIR. Additionally, a uniform offset of +15 was added to all intensity values poststandardization to avoid negative values. Default values were used for all other extraction parameters.

**Figure 1.**
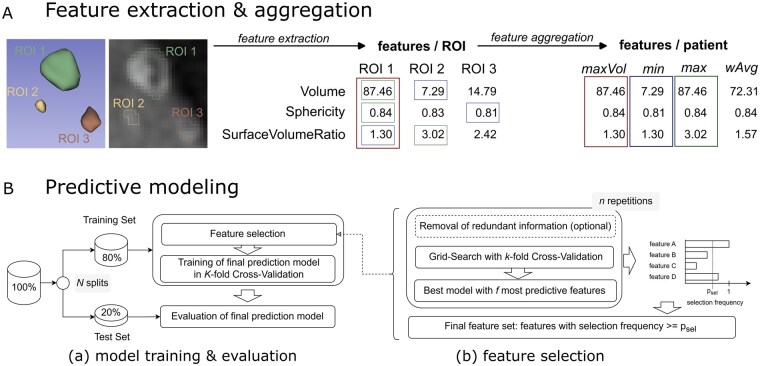
Overview of the methods used in this study: (A) Radiomics feature extraction and aggregation: After segmentation of the regions of interest (ROIs), radiomics features were extracted from each ROI across four MRI modalities (T1, T1c, T2, FLAIR). To enable statistical analysis and model training, features from multiple ROIs per patient were aggregated, resulting in a single feature set per patient, per modality, for each of four aggregation strategies: volume-weighted average (wAvg), minimum (min), maximum (max), and largest-volume ROI (maxVol). While only a subset of shape features is visualized here, the same aggregation procedure was applied to all 107 extracted radiomics features. (B) Predictive modeling workflow: (a) Statistical evaluation was based on repeated (N = 20) model training and testing cycles. In each repetition, the dataset was randomly split into a training and a test set. Feature selection and model training were performed on the training set, while performance was evaluated on the independent test set. (b) Feature selection involved a repeated (n = 20) grid-search cross-validation procedure. Only features that were consistently selected across the n repetitions were retained for inclusion in the final model training phase.

For each patient and sequence, radiomics features were aggregated across multiple ROIs using four different strategies: (i) volume-weighted average (wAvg), (ii) minimum value (min), (iii) maximum value (max), and (iv) value from the largest-volume ROI (maxVol). Two supplementary features, the number of ROIs per patient and the total ROI volume, were also included. This resulted in a total of 4 × (107 + 2) = 436 features per patient and MRI sequence. Figure 1A illustrates the feature extraction and aggregation workflow.

### Radiomics and Statistical Analysis

Two separate analyses were conducted to evaluate the predictive value of MRI radiomics for tumor recurrence and to explore the relationship between aggregated radiomics features (ie, wAvg, min, max, or maxVol) and this clinical endpoint. Both analyses were implemented in Python using the Scikit-learn^10^ (v 1.6.1) library for machine learning, and the stats package of the SciPy (v 1.15.2) library for statistical testing.

#### Multivariable predictive modeling

The first analysis focused on multivariable modeling to predict FET-PET-confirmed tumor recurrence using radiomics features from FLAIR and T1c MRI sequences. Logistic regression (LR) models with Elastic-Net regularization were trained, with the area under the receiver operating characteristic curve (ROC AUC) as the primary performance metric. The Elastic-Net penalty was used to encourage sparsity and reduce overfitting by promoting models with fewer, more informative features. In addition, the removal of redundant information by discarding highly correlated (Spearman correlation > 0.8) features prior to feature selection was evaluated. All four feature aggregation strategies were systematically evaluated. Since the outcome classes were nearly balanced (recurrence: 21, no recurrence: 17), no class imbalance compensation techniques were applied during model training.

A thorough statistical evaluation approach was chosen to obtain robust performance estimates of predictive models despite the absence of an independent evaluation cohort in this study (Figure 1B). N repetitions of random stratified splits into 80% training/validation and 20% internal testing sets were evaluated independently. In each repetition, feature selection followed a three-step process. First, an optimal subset of features was identified using k-fold grid-search cross-validation (CV) on the training data. This step was repeated n times with different CV seeds, resulting in multiple models with up to f features. Second, the selection frequency of each feature across all n repetitions was computed as the number of times a feature had been selected divided by the number of repetitions n. Features with a selection frequency close to 1 were considered robust predictors, while those with low frequencies were deemed unlikely to provide predictive value. Third, features meeting a minimum selection frequency threshold p_sel were included in the final feature set. A model with the final feature set was then retrained on the entire training/validation set in k-fold CV and evaluated on the independent internal test set to assess its generalizability.

To ensure consistency, features were standardized via z-score standardization in each of the N repetitions based solely on the training split, and this scaling was applied to the corresponding test split.

The hyperparameters N = 20 splits, n = 20 feature selection repetitions, k = 3 folds, and p_sel = 0.5 were chosen based on prior experience and dataset size. The optimal number of features f was determined by evaluating multiple values (5, 10, 15, 20) and selecting the one maximizing average CV training performance.

To assess model robustness, 95% confidence intervals (CIs) were computed using bootstrapping. Specifically, l = 10 000 samples were drawn from the m = 20 internal test AUCs and the m = 60 CV AUCs (ie, 20 repetitions × 3 folds), thus creating l bootstrap samples of m performance estimates each. The mean of each bootstrap sample yielded l estimates of the mean AUC, from which the 95% CI was calculated (2.5th-97.5th percentiles). Performance results are reported as mean AUC [95% CI], and a model was considered significantly predictive if its 95% CI excluded 0.5.

#### Univariable statistical analysis

The second analysis aimed to identify individual radiomics features that were significantly associated with FET-PET-confirmed recurrence. First, redundant features were removed by eliminating highly correlated variables (Spearman ρ > 0.8), resulting in reduced sets of n weakly correlated features. For each remaining feature, the distributions in recurrence vs non-recurrence groups were compared using univariable statistical tests. If a feature was normally distributed (as determined by the Shapiro-Wilk test), an independent Student’s t-test was applied; otherwise, a Mann-Whitney U-test was used. The global significance threshold (*P* < .05) was adjusted using Bonferroni correction to account for the n-multiple testing. This analysis was performed separately for each combination of MRI sequence and aggregation approach.

## Results

### Patient Population

A total of 38 patients (37% female) aged (mean/median/range) 47.4/51/[20, 75] years at diagnosis were included in this study. The primary glioma was IDH mutant in 18 patients (47%), and IDH-wildtype in 20 patients (53%). The time from primary surgery to radiological suspicion of recurrence was (mean/median/range) 27.7/20/[0, 86] months. The time interval between the MRI showing suspected recurrence and the FET PET examination was 22.0/16.5/[0, 79] days. Although patient demographics, treatment characteristics, and primary tumor characteristics were not fully balanced between the recurrence (n = 21) and non-recurrence (n = 17) cohorts, no statistically significant differences were observed between the groups in these variables. However, among patients with confirmed tumor recurrence, IDH wild-type tumors were more frequent compared to the non-recurrence group (67% vs 35%, *P* = .05). See Table A1 in the Appendix for further details.

Across all 38 patients, 59 imaging anomalies were identified on T1c MRI and segmented as lesion ROIs with (mean/median/range) volume of 1.920/0.119/[0.001, 29.088] ccm. In 15 patients, multiple ROIs (ranging from 2 to 3 per patient) were identified. Figure A3 in the Appendix illustrates the distribution of ROI volumes.

### Multivariable FLAIR and T1c Radiomics Signatures Are Predictive on the Internal Test Set

The development of radiomics signatures by predictive modeling was investigated using FLAIR and T1c MRI sequences, incorporating all extracted radiomics features and feature aggregation approaches. Among the aggregation approaches, aggregation by the minimum value (min) across lesions and by the maximum volume lesion (maxVol) yielded significantly predictive models for both FLAIR and T1c derived features with (internal) test AUCs of 0.71 [0.63, 0.78], 0.68 [0.59, 0.76] (min) and 0.65 [0.55, 0.73], 0.63 [0.55, 0.70] (maxVol), respectively. Table 1 summarizes these findings.

**Table 1.** Predictive performance (mean [95% CI] of ROC-AUC) of radiomics signatures derived from MR FLAIR and T1c modalities and using different aggregation approaches

MR modality	Aggregation	AUC (CV)	AUC (test)
FLAIR	**min**	0.89 [0.86, 0.92]	**0.71 [0.63, 0.78]**
	max	0.88 [0.85, 0.90]	0.59 [0.50, 0.68]
	wAvg	0.80 [0.76, 0.83]	0.47 [0.39, 0.54]
	**maxVol**	0.90 [0.87, 0.92]	**0.65 [0.55, 0.73]**
T1c	**min**	0.88 [0.85, 0.91]	**0.68 [0.59, 0.76]**
	max	0.82 [0.79, 0.86]	0.56 [0.46, 0.65]
	wAvg	0.79 [0.76, 0.83]	0.53 [0.45, 0.62]
	**maxVol**	0.89 [0.85, 0.91]	**0.63 [0.55, 0.70]**

Aggregation approaches resulting in statistically significant predictive performance on the internal test are printed in bold.

For both FLAIR and T1c-derived radiomics, Shape Sphericity was the only feature consistently selected across all N data splits and model repetitions for both min and maxVol aggregation strategies. This was followed by the frequent selection of various texture features. Sphericity is a dimensionless metric quantifying the roundness of an ROI with values ranging from 0 to 1, where 1 represents a perfect sphere. Table 2 lists the most frequently selected features under the min-aggregation strategy, along with their corresponding selection frequency.

**Table 2.** Most important radiomics features for min-aggregation models from Table 1

FLAIR (min-aggregation)		T1c (min-aggregation)	
Feature group/name	Selection frequency [%]	Feature group/name	Selection frequency [%]
Shape/Sphericity	100	Shape/Sphericity	100
Texture/GLCM IMC1	75	Texture/NGTDM Busyness	45
Intensity/Kurtosis	70	Texture/GLDM Large Dependence Low Grey Level Emphasis	45
Texture/GLCM IMC2	60	Texture/GLCM Correlation	40
Texture/NGTDM Strength	50	Texture/GLCM IMC2	30
Texture/GLCM Cluster Prominence	50		
Intensity/RMS	45		
Texture/GLDM Large Dependence High Grey Level Emphasis	40		
Intensity/Skewness	40		
Intensity/Mean	35		

The selection frequency column indicates how often the respective feature was included in the models built across N = 20 (Figure 1B(a)) splits. Only features included in at least 30% of the models are shown.

### Shape Features Drive Recurrence Prediction

To investigate which feature groups are most relevant for recurrence prediction, the same modeling approach was applied independently to each feature group (shape, intensity, texture), focusing on the min-aggregation approach which had previously yielded significantly predictive models for both FLAIR and T1c sequences. For both modalities, shape features alone resulted in significantly predictive models, achieving internal test AUCs of 0.78 [0.70, 0.85] for FLAIR and 0.76 [0.70, 0.81] for T1c, see Table 3. The most influential shape features were Sphericity, Volume (voxel- or mesh-based), and Surface Volume Ratio, with feature selection frequencies ranging from 70% to 100%. Surface Volume Ratio represents the ratio of the ROI surface to its volume; lower values indicate more compact shapes. Individually, lower values of min-aggregated Sphericity and Surface Volume Ratio but higher values of min-aggregated Volume were associated with glioma recurrence. In addition, min-aggregated texture features derived from FLAIR alone also yielded significantly predictive models.

**Table 3.** Predictive performance (mean [95% CI] of ROC-AUC) of multi-variable radiomics signatures developed exclusively from specific feature groups (shape, intensity, texture) using min-aggregation

MR modality	Feature group	AUC (CV)	AUC (Test)
FLAIR	**Shape**	0.85 [0.82, 0.87]	**0.78 [0.70, 0.85]**
	Intensity	0.66 [0.62, 0.70]	0.57 [0.50, 0.64]
	**Texture**	0.81 [0.78, 0.84]	**0.58 [0.51, 0.65]**
T1c	**Shape**	0.83 [0.80, 0.87]	**0.76 [0.70, 0.81]**
	Intensity	0.57 [0.53, 0.62]	0.39 [0.31, 0.47]
	Texture	0.81 [0.77, 0.85]	0.51 [0.43, 0.58]

Statistically significant results on the internal test are highlighted in bold.

### Univariable Feature Analysis Confirms Shape Sphericity Significantly Associated With Recurrence

To further examine the association between individual radiomics features and tumor recurrence, univariable feature selection was performed for each aggregation strategy and imaging modality, following the prior removal of redundant features (Spearman correlation > 0.8). Only features with Bonferroni-corrected *P*-value ≤ .05 were retained as statistically significant. Among all evaluated features, Shape Sphericity, aggregated using either min and maxVol approaches, was the only feature consistently selected in both FLAIR and T1c modalities, aligning with its status as the highest-ranking feature in Table 2. Further details are provided in Table A4 in the Appendix.

**Figure 2.**
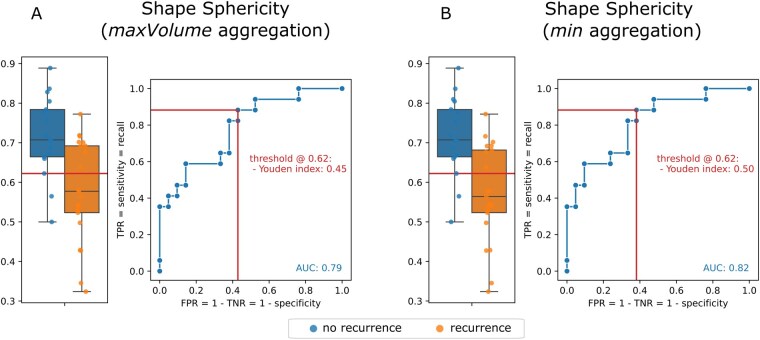
Distribution of feature values grouped by outcome (no recurrence vs recurrence) and corresponding ROC curves for Shape Sphericity: (A) maxVol-aggregated, AUC = 0.79, and (B) min-aggregated, AUC = 0.82. Optimal classification thresholds were determined by maximizing the Youden index.

When evaluated individually, Sphericity enabled discrimination between patients with and without recurrence, achieving ROC AUCs of 0.82 (min aggregation) and 0.79 (maxVol aggregation), see Figure 2.

**Figure 3.**
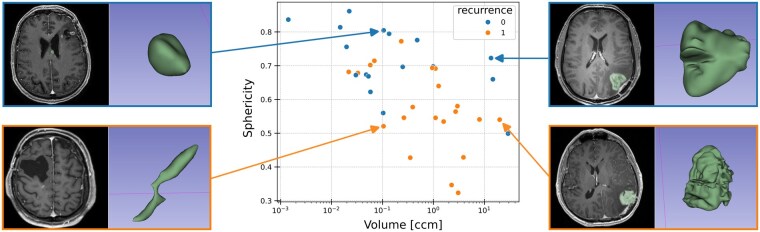
Examples of ROIs in function of volume and min-aggregated sphericity feature values, as well as recurrence outcome. Most high-sphericity ROIs are associated with non-recurrence, and vice versa, across different volumes.

Figure 3 illustrates the shapes of selected ROIs with high/low Sphericity and different recurrence outcomes over a range of ROI volumes. Very high sphericity was predominantly observed in smaller-volume ROIs, whereas very low sphericity was more common in larger-volume ROIs. Across different ROI sizes, most high-sphericity ROIs (nearly spherical with smooth surfaces) were associated with non-recurrence, while the majority of low-sphericity ROIs (markedly non-spherical with highly structured surfaces) were linked to recurrence.

## Discussion

In this study, we investigated the value of radiomics in the postoperative setting of adult type gliomas at the time of neuroradiological suspicion of recurrence on MRI. FLAIR and T1c imaging were used to develop radiomics signatures via predictive modeling, exploring various radiomics feature groups and aggregation approaches. Aggregation by the minimum value (min) across ROIs and selection by maximum ROI volume (maxVol) produced significantly predictive models for both modalities.

Using the min-aggregation approach, multivariable radiomics models based on shape features achieved strong predictive performance on the internal test set, with AUCs of 0.78 [0.70, 0.85] for FLAIR and 0.76 [0.70, 0.81] for T1c. Key shape features driving these models included Sphericity, Volume (voxel- or mesh-based), and Surface Volume Ratio. Univariable analysis confirmed that Shape Sphericity, aggregated by min and maxVol, distinguished recurrence. Although multiple ROIs were identified in 15 of 38 patients, the number of T1c ROIs was not significantly associated with recurrence (*P* = .64).

Our radiomics analysis focused exclusively on newly detected contrast-enhancing tissue on follow-up MRI, either in a new location or contiguous with previously existing residual tumor, rather than including all tissue suspected to be tumor on follow-up MRI. This distinction is critical, as residual enhancing or suspicious FLAIR tissue may persist from prior treatment. This approach differs from previous studies, such as Ren et al,^5^ who delineated all contrast-enhancing tissue regardless of new appearance and better reflects typical clinical follow-up scenarios.

We observed comparable importance and predictive value for shape features extracted from both FLAIR and T1c imaging. This finding is expected, as the primary lesion contours were delineated on T1c images and then transferred to FLAIR images via co-registration; minor differences between the modalities arise from their distinct image resolutions. In both cases, the shape features characterize the appearance of imaging anomalies visible on T1c scans. Interpreting the dominant shape features under the min-aggregation approach is complex. Figure 3 illustrates the relationship between min-aggregated Sphericity and Volume: small, elongated ROIs or large ROIs with irregular, spiky surfaces were associated with recurrence. Similar patterns, as reported in Table 3 for min-aggregation, were also observed for maxVol-aggregation.

This finding aligns with the often-irregular shapes of recurrent tumors observed on MRI in clinical routine. Very recently, a study in IDH-wildtype gliomas demonstrated the importance of shape features, showing that patients with tumors of higher sphericity and elongation (prior to surgery) had more than twice the survival time compared to those with greater maximum 3D diameter, surface area, axis lengths, and tumor volume.^11^ While biological interpretation of radiomics features is desirable, it is not always necessary for establishing their clinical utility.^12^

In contrast to most published predictive glioma radiomics models that used pre-operative imaging to characterize the unresected tumor, this study focused on prediction from postoperative scans based on new suspicious contrast-enhancing lesions in T1c. For example, radiomics has been applied previously to predict recurrence in high-grade gliomas,^13^ including glioblastoma,^14^ to differentiate therapeutic effects such as radiation necrosis^15-17^ and pseudoprogression in glioblastoma,^18^ and to identify residual tumor after surgery. A recent meta-analysis reviewing 27 studies on predicting therapeutic effects highlighted that, although radiomics shows promise, challenges remain related to study quality, publication bias, and significant heterogeneity across investigations.^19^ This heterogeneity complicates direct comparisons and hampers the reproducibility of radiomics signatures between studies.

While many prior studies have focused predominantly on patients with a single histopathological subtype, often glioblastoma,^20-26^ our study reflects a more common clinical scenario where glioma recurrence is suspected radiologically, and tumor board decisions must be made across a spectrum of primary glioma histologies. Accordingly, our cohort included patients with diverse glioma types, see Table A2 in the Appendix for details. Investigating such a heterogeneous population may broaden the clinical applicability of radiomics analyses beyond a single glioma subtype, enhancing its practical utility in routine neuroradiology practice.

In this context, the theoretical differences between IDH-wildtype (wt) and IDH-mutant gliomas are highly relevant. IDH-wt tumors typically exhibit more aggressive, metabolically active, and infiltrative growth patterns compared to the often more circumscribed IDH-mutant lesions. Indeed, in our dataset IDH-wt tumors demonstrated significantly larger volumes and lower (min-aggregated) sphericity (*P* = .019) compared to IDH-mutant cases, mirroring the known aggressive imaging phenotype of wildtype gliomas. Crucially, while (min-aggregated) sphericity was associated with IDH status (*P* = .019), it remained more closely aligned with the FET-PET-confirmed recurrence status (*P* = .0004). This suggests that this shape feature captures morphological changes specific to the recurrent environment, which is often a complex mixture of proliferating tumor cells, reactive tissue, and treatment-related necrosis. While digitized photomicrographs were not available to provide direct histologic correlation for this retrospective cohort, our results suggest that MRI-based shape features, particularly sphericity, can serve as a noninvasive proxy for the underlying metabolic and pathological complexity that defines true recurrence. Given our cohort size (n = 38), further disentangling the independent predictive value of other shape features from molecular confounders remains a limitation to be addressed in larger studies.

In this study, we included patients diagnosed with glioma who had available postsurgical MRI scans, without restricting the time interval between resection and imaging, to better reflect clinical reality in neuroradiology. Although this interval varied between 0 and up to 86 months, it was not significantly associated with tumor recurrence (*P* = .43; see Table A1 in the Appendix). Importantly, suspected glioma recurrence was confirmed by subsequent surgery in 17 of 21 patients (81%). Cohorts with histopathologically confirmed glioma recurrence remain relatively rare in the radiomics literature.^25^^,^^27^^,^^28^

While our study demonstrates how the selected radiomic features correlate with IDH status, further integration with broader molecular genomics (radiogenomics) remains a compelling future direction. Such a multi-omics approach could potentially bridge the gap between macroscopic imaging phenotypes and underlying molecular profiles.

### Limitations

Although this study benefited from high-quality annotations, including manually curated segmentations, FET-PET-based ground truths, and histopathological confirmation of recurrence status in 21 of 38 patients (55.3%), several limitations may affect the generalizability of our findings. The study cohort was relatively small, comprising only 38 patients from a single center. Due to the retrospective nature of the analysis, pathological classifications were primarily performed according to the WHO CNS tumor classifications of 2007 and 2016, with IDH status updated according to the 2021 WHO classification.^29^

While our modeling approach ensures robustness within the current dataset, the generalizability of the developed radiomics signatures to other populations remains untested due to the lack of an external validation cohort.

Furthermore, we did not assess the impact of inter-observer variability in contouring the suspected tumor tissue, which may influence radiomics results.

Despite promising results, radiomics prediction models still require validation and better workflow integration before clinical use. While many studies aim to predict recurrence patterns, such models have not yet influenced clinical decisions due to variability, reproducibility issues, and the inherent unpredictability of tumor evolution.

## Conclusion

In this study, we addressed a common clinical scenario: guiding management decisions for individual patients when glioma recurrence is suspected on routine postoperative MRI. This particular context has been underexplored in radiomics research, largely due to the challenges of defining reliable regions of interest on postsurgical images. Leveraging two widely available MRI sequences, FLAIR and contrast-enhanced T1 (T1c), we developed radiomics models that demonstrated promising performance for predicting recurrence, achieving an AUC of 0.78 [0.70, 0.85] (FLAIR) and 0.76 [0.70, 0.81] (T1c) on an internal test set.

When both radiological evaluation and radiomics signatures indicate suspicion of recurrence, our approach could provide stronger justification for proceeding with more costly and resource-intensive examinations such as FET-PET imaging. Conversely, if the radiomics model suggests a low likelihood of recurrence, this may support a strategy of close routine monitoring with earlier follow-up MRI, potentially conserving financial and personnel resources.

In summary, we identified a group of common radiomics features characterizing the shape of imaging anomalies on T1c imaging that were significantly predictive of tumor recurrence. The dominant features, Sphericity, Volume (voxel- or mesh-based), and Surface Volume Ratio, were consistently selected in 70% to 100% of model iterations.

After prospective confirmation in a larger multi-institutional cohort, external validation, and assessment of annotation stability, these radiomics findings have the potential to enhance clinical decision-making workflows in neuro-oncology.

## Supplementary Material

vdag143_Supplementary_Data

## Data Availability

The data utilized for this study (patient images, outcome information, and clinical covariates), as well as data generated by the study (ROI annotations, radiomics feature values), are not currently publicly available. Software code for data analysis will be made available upon request. The configuration choices for radiomics feature extraction are included in the Supplementary Materials.
